# Multi-Task Partial Offloading with Relay and Adaptive Bandwidth Allocation for the MEC-Assisted IoT

**DOI:** 10.3390/s23010190

**Published:** 2022-12-24

**Authors:** Hafiz Hasnain Imtiaz, Suhua Tang

**Affiliations:** Department of Computer and Network Engineering, The University of Electro-Communications, 1-5-1 Chofugaoka, Chofu 182-8585, Tokyo, Japan

**Keywords:** multi-access edge computing, partial offloading, relay, resource allocation, latency optimization

## Abstract

The fifth-generation (5G) wireless network is visualized to offer many types of services with low latency requirements in Internet of Things (IoT) networks. However, the computational capabilities of IoT nodes are not enough to process complex tasks in real time. To solve this problem, multi-access edge computing (MEC) has emerged as an effective solution that will allow IoT nodes to completely or partially offload their computational tasks to MEC servers. However, the large communication delay at a low transmission rate for nodes far from the access point (AP) makes this offloading less meaningful. This paper studies joint multi-task partial offloading from multiple IoT nodes to a common MEC server collocated with an AP, and it uses relay selection to help nodes far from the AP. The computation time of all tasks is minimized by adaptive task division and resource allocation (bandwidth and computation resource), and it is solved with an evolutionary algorithm. The simulation results confirm that the proposed method with both relay selection and adaptive bandwidth allocation outperforms the methods with neither or only one function.

## 1. Introduction

Recent developments in wireless communications and sensing techniques have promoted the Internet of Things (IoTs). Initially, IoT nodes were used to realize simple sensing tasks, such as measuring temperature or moisture, and most IoT nodes have limited resources for computation, storage, and battery life.

The emergence of AI techniques enables more advanced tasks, such as surveillance and intrusion detection, which heavily rely on the deep learning technique to improve their accuracy and require more complex processing at IoT nodes [1]. Although current CPUs on IoT nodes have become more powerful, it is still difficult to process computation-intensive tasks in real time, and the large power consumption will quickly deplete the battery of an IoT node [2].

To overcome the above problem, in the last few decades, mobile cloud computing (MCC) has been suggested to assist IoT nodes with low computational capabilities by exploiting the abundant processing power and storage in the cloud. Specifically, IoT nodes collect and send data to cloud servers for processing, and the control decision based on the processing result is sent back to actuators collocated with the sensors. This helps to reduce the processing delay, but the long distance between IoT nodes and the cloud servers may bring vast communication latency, especially when the sensing data are multimodal with a large size, which makes it hard to meet the criteria of delay-sensitive applications [3,4].

A new technology, named multi-access edge computing (MEC), has been proposed to meet the strict delay requirement. An MEC server is deployed at the cloud edge near IoT nodes. With more computation resources and storage than IoT nodes, an MEC server can help IoT nodes to process computation-intensive tasks, while its short distance to IoT nodes also helps to suppress the communication delay [5,6,7].

The computation task offloading to MEC servers has attracted much attention in recent years. It is generally divided into two types: binary offloading and partial offloading [8]. In binary offloading, a node either computes a task locally or totally offloads it to an MEC server. Binary offloading has some drawbacks, such as unnecessary consumption of energy and time for the computation that is ultimately offloaded. Another problem is that when fully offloading a task, its execution may be delayed because of bandwidth limitations. In contrast, a partial offloading scheme divides a task into two segments. One segment of the task is executed on the node locally, and the other segment is offloaded to an MEC server. Partial offloading efficiently utilizes both the local and MEC resources. With good splitting of a task between local processing and MEC offloading, partial offloading can efficiently minimize the task execution time, and it is a promising technique for the IoT [9,10,11,12].

IoT nodes usually are connected with MEC servers by wireless communications. The wireless channel is susceptible to path loss and channel fading, which may greatly degrade the transmission rate. When a task has a very large data set, but the transmission rate between the IoT node and the MEC server is low, the communication itself takes a great deal of time, and the effect of partial offloading will be degraded. Relaying was exploited in [13] to alleviate this problem. In a network with multiple IoT nodes sharing the same spectrum resource, equally allocating bandwidth is a reasonable policy. However, the volume of data generated by each task is time-variant, and it is necessary to dynamically allocate the resource to improve the spectrum efficiency. Adaptive resource allocation was studied in [14] for binary offloading.

This paper studies joint multi-task partial offloading from multiple IoT nodes to a common MEC server collocated with an AP, aiming to address the heterogeneity in transmission rates. To this end, an algorithm named Partial Offloading with Relay and Adaptive Bandwidth Allocation (PORAB) is proposed to minimize the execution time of all tasks. PORAB is an evolutionary algorithm. It initiates a population of candidates. In each generation, it drops the lowest part of the population and generates new candidates using the characteristics of highly fit candidates.

Our contribution is twofold:

- We investigate the heterogeneity in transmission rates and suggest using a relay to help nodes far from the AP and combine it with adaptive bandwidth allocation to achieve fine-grained resource allocation.
- We propose an evolutionary algorithm for joint optimization of radio resources (bandwidth) and computation resources. This not only improves the performance of nodes using relays but also reduces the delay of other nodes.

The proposed method is evaluated with MATLAB simulations, and the impacts of the number of tasks, size, and the volume of data on the task execution time are analyzed. The simulation results confirm that the proposed method with both relay selection and adaptive bandwidth allocation outperforms methods without the functions or with only one function.

The remainder of the paper is divided into five sections. Related works are reviewed in Section 2. The system model, including a relay communication model and problem formulation, is presented in Section 3. Section 4 describes the proposed method, while the simulation analysis and evaluation are presented in Section 5. Finally, Section 6 concludes this paper.

## 2. Related Work

Partial offloading is especially suitable for applications with low latency requirements. Some work has been performed in this field. In [15], Wang et al. adopted partial offloading to enhance the computation offloading performance. Le et al. [16] and Ren et al. [17] investigated resource allocation and data segmentation in order to minimize latency by considering a partial offloading scheme. In [13], Cao et al. studied the concept of cooperative communication and considered three nodes, with one of them working as a relay. They considered both binary and partial offloading schemes and jointly optimized the resource allocation and computation for both the relay and nodes. For delay-sensitive applications, in [18], Chen et al. proposed a green parallel online offloading algorithm based on Lyapunov optimization. Multiple devices can offload their data to MEC servers in parallel, and a joint optimization problem is formulated. The algorithm adaptively selects a server for a device according to its channel state and current location. In [19], Katayama et al. considered an MEC system with three types of servers, formulated an optimization problem for resource allocation, and proposed a heuristic algorithm to minimize the total latency. Zhang et al. in [20] investigated the task offloading problem of multiple nodes for an MEC system and jointly optimized the offloading delay and energy consumption. In [21], Huang et al. optimized the resource allocation and task offloading decision by adopting a binary offloading policy and solved it with a deep reinforcement learning-based framework. Meanwhile, in [22], Chen et al. proposed a software-defined network and minimized the task offloading time to MEC servers via a binary offloading scheme. The problem of reducing energy utilization was investigated in [23] to minimize the overloading battery usage by using the wireless power transfer to nodes and the partial offloading to MEC servers. In [24], a Lyapunov optimization problem was formulated with the objective of jointly reducing the energy consumption and task execution time, and the authors proposed a secure offloading framework, combining edge computing and cloud computing to achieve optimal task offloading where the MEC servers provide low-latency computation assistance while the MCC servers offer powerful computation.

In [25], considering both energy usage and time delay, joint multi-task partial offloading was formulated to achieve on-demand offloading, and the authors stated that partial offloading outperformed binary offloading. In [26], Guo et al. stated that the best resource allocation can improve the energy efficiency, latency, and computational capabilities of the nodes. The above statement was proven by Mahmood et al. in [27], who proposed to minimize the duration of multiple tasks through excellent placement of computational assets’ power supplies and optimal task segmentations. Although only a part of a task is transmitted to an MEC server in partial offloading, its transmission time is non-negligible and may be long at a low transmission rate for nodes far from the access point (AP). This problem, however, was not taken seriously in previous works.

In recent years, intelligent offloading has attracted much attention. In [28], Qu et al. proposed a deep meta reinforcement learning-based offloading algorithm which integrates various parallel deep neural networks with Q-learning to achieve efficient offloading decision making. The purpose is to reduce the latency and energy computation. In [29], the concept of the wireless power mobile edge cloud (WPMEC) with partial offloading was studied, where the MEC is integrated with wireless power transfer (WPT) at the AP. In [23], the authors confirmed that a partial offloading scheme outperformed the binary counterpart.

This paper also adopts the policy of partial offloading. Compared with previous work in this field, this paper considers the potential poor transmission rate and time-variant data size in each task. Accordingly, relay selection is used to help nodes far from the AP, and adaptive bandwidth allocation is exploited to address both the heterogeneity in transmission rates and the variation in data volumes of tasks. We notice that relay was exploited in [13], where a relay node also helped computation, and adaptive resource allocation was studied in [14] for binary offloading. In comparison, this paper combines both to achieve better performance for partial offloading.

## 3. System Model

In this paper, we consider a wireless cell with an AP and *N* IoT nodes. Nodes are randomly distributed in the coverage of the AP. They have low computational capabilities and cannot process power-hungry and computation-intensive tasks in real time. Therefore, an MEC system is deployed, collocated with the AP to deliver computational services to the nodes on demand.

We assume each node has only one task. The task at node *n* has a data size $s_{n}$ and requires $c_{n}$ cycles to process. The processing capability of node *n* is $f_{n}^{L}$ cycles per second, and the computational capability of the MEC server is $f^{max}$ cycles per second. The computation resource allocated at the MEC server to task *n* is $f_{n}^{M}$ cycles per second. The overall bandwidth is *B*, and the percentage of bandwidth for task *n* is $b_{n}$, which is decided according to the signal strength and other factors. To fully exploit the computation resource for both IoT nodes and the MEC server, this paper adopts a partial offloading policy. We denote the percentage of local computations for task *n* as $\alpha_{n}$, and the remaining part ($1-\alpha_{n}$) will be offloaded to the MEC server. The main notations are summarized in Table 1.

**Figure 1:**
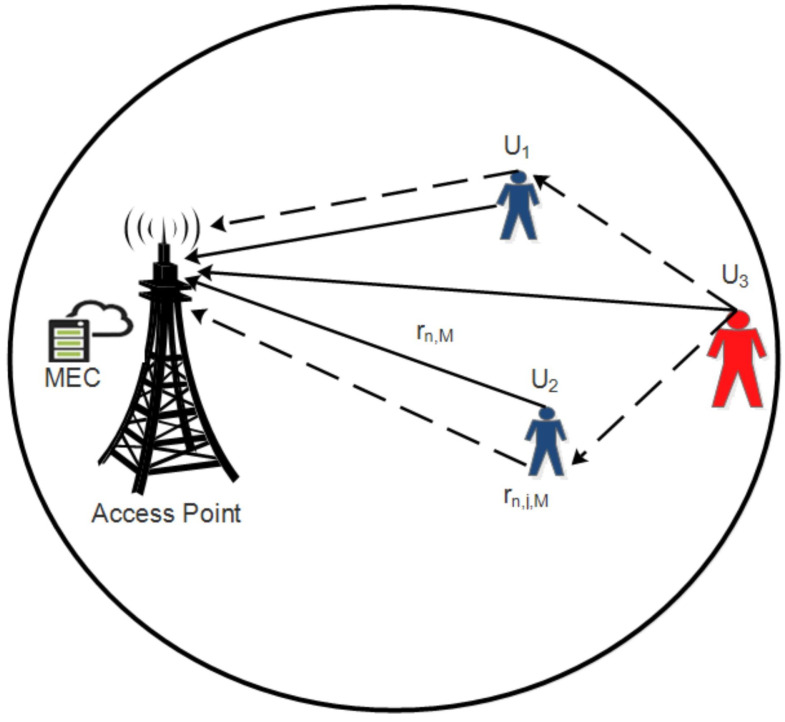
System model of relay-based multi-task partial offloading in multi-access edge computing.

When a node offloads its computation task to the MEC server, the delay consists of three parts: (1) time for transmitting data to the MEC server via the AP, (The link between the MEC server and the AP uses high-speed Ethernet, and the transmission time is neglected.) (2) time for processing the data at the MEC server, and (3) time for transmitting the result from the MEC server back to the node. Here, it is assumed that the size of the result is negligibly small. The time in (1) depends on the transmission rate, which further depends on the distance between the nodes and the AP. As shown in Figure 1, the blue nodes (U1 and U2) are close to the AP, and they can offload their data to the MEC server by direct transmission at a high rate. However, the red node (U3) is far from the AP and near the cell edge. Its direct transmission rate is low, which will cause a large delay. In this paper, we use the relay method to solve this problem and adaptive bandwidth allocation to fine-tune the transmission rate.

### 3.1. Relay Model and Adaptive Bandwidth Allocation

According to the Shannon theory, the rate for direct transmission from node *n* to the AP is

(1) $$r_{n,M}=b_{n}B\log_{2}\left(1+\frac{p_{n}g_{n}}{\sigma^{2}}\right)$$

where $p_{n}$ is the transmission power of node *n*, $g_{n}$ represents the channel gain between node *n* and the AP, $\sigma^{2}$ represents the noise power, $b_{n}\left(0\leq b_{n}\leq 1,\sum_{n}b_{n}=1\right)$ represents the percentage of bandwidth allocated for node *n*, and *B* represents the total bandwidth of the system.

**Figure 2:**
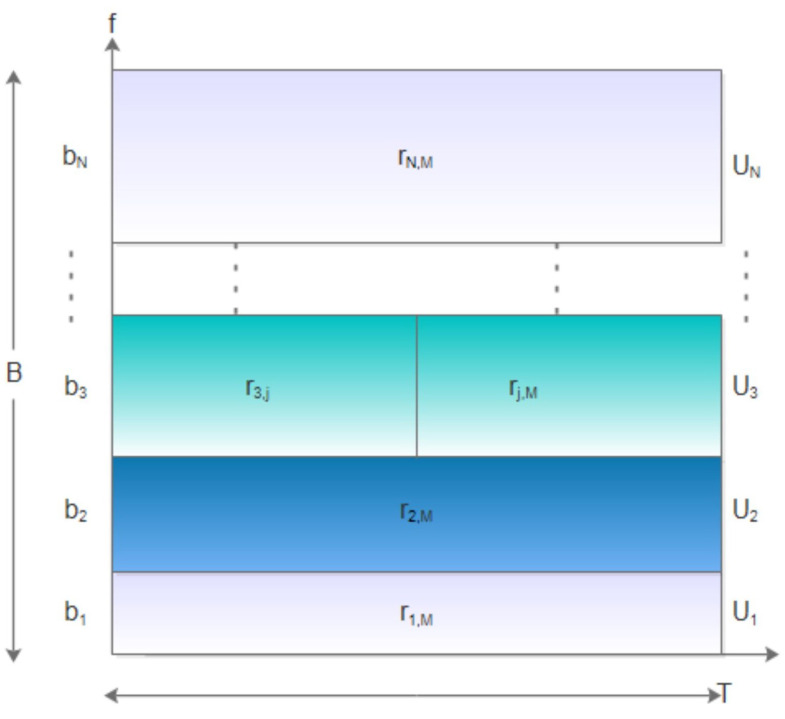
Resource allocation for nodes and relay.

When node *n* transmits its task data via a relay node *j*, it shares its bandwidth with the relay node, and they transmit in different periods. The whole transmission involves two rates: $r_{n,j}$ from node *n* to relay node *j* in the first period and $r_{j,M}$ from relay node *j* to the AP in the second period, as shown in Figure 2.

The period length is inversely proportional to the transmission rate, and the ratio between the first and second period lengths is $r_{j,M}:r_{n,j}$. Then, the equivalent rate between node *n* and the AP, using relay *j*, is

(2) $$r_{n,j,M}=\frac{1}{\frac{1}{r_{n,j}}+\frac{1}{r_{j,M}}}=\frac{r_{n,j}\cdot r_{j,M}}{r_{n,j}+r_{j,M}}.$$

The relay selection problem is finding a relay node *j* that maximizes the rate

(3) $$j^{*}=\arg\max_{j}r_{n,j,M}.$$

If $r_{n,j^{*},M}$ is greater than $r_{n,M}$, then the relay node $j^{*}$ should be used; otherwise, direct transmission should be used. Then, the overall rate from node *n* to the AP is

(4) $${\hat{r}}_{n}=\max\left(r_{n,M},r_{n,j^{*},M}\right).$$

### 3.2. Computation Time

Node *n* locally processes a part ($\alpha_{n}$) of its task, and the required time is

(5) $$T_{n}^{L}=\frac{\alpha_{n}c_{n}}{f_{n}^{L}}.$$

Node *n* offloads $1-\alpha_{n}$ of its task to the MEC server. The transmission time is $\frac{\left(1-\alpha_{n}\right)s_{n}}{{\hat{r}}_{n}}$. At the MEC server, the processing time is $\frac{\left(1-\alpha_{n}\right)c_{n}}{f_{n}^{M}}$. Then, the overall edge computation time for task *n* is

(6) $$T_{n}^{M}=\frac{\left(1-\alpha_{n}\right)s_{n}}{{\hat{r}}_{n}}+\frac{\left(1-\alpha_{n}\right)c_{n}}{f_{n}^{M}}.$$

The overall computation time of task *n* is equal to $\max\{T_{n}^{L},T_{n}^{M}\}$.

### 3.3. Problem Formulation

As mentioned above, this paper aims to minimize the computation times of all tasks by relay selection and optimal allocation of resources (such as bandwidth, task division between local computation and offloading, and allocation of computation resources at the MEC server for all tasks).

Mathematically, we define our joint optimization problem as follows:

(7a) $$\begin{array}{cc} \min_{\alpha_{n},b_{n},f_{n}^{M}} & \max_{n}\left(\frac{\alpha_{n}c_{n}}{f_{n}^{L}},\left(1-\alpha_{n}\right)\left(\frac{s_{n}}{{\hat{r}}_{n}}+\frac{c_{n}}{f_{n}^{M}}\right)\right), \end{array}$$

(7b) $$\begin{array}{cc} C_{1}: & \sum_{n=1}^{N}f_{n}^{M}\leq f^{max},f_{n}^{M}\geq 0, \end{array}$$

(7c) $$\begin{array}{cc} C_{2}: & \sum_{n=1}^{N}b_{n}\leq 1,b_{n}\geq 0, \end{array}$$

(7d) $$\begin{array}{cc} C_{3}: & \;0\leq \alpha_{n}\leq 1. \end{array}$$

The objective of Equation (7a) is to minimize the maximal processing time of all tasks. $C_{1}$ in Equation (7b) states that the sum of the computation resources allocated to all tasks must be less than the maximum computational capability of the MEC server. $C_{2}$ in Equation (7c) ensures that the sum of the allocated bandwidth for all nodes must be less than the total bandwidth. $C_{3}$ in Equation (7d) indicates the lower and upper bounds of the task division.

## 4. Proposed Method

### 4.1. Optimal Calculation of $\alpha_{n}$

As can be seen in Equation (7a), our objective function, having three decision variables, is non-convex in nature. We optimally find the value of the task division $\alpha_{n}$ by fixing the values of the remaining parameters, which makes it easier to find the solution.

In our optimization problem, time delay consists of the local computation time $T_{n}^{L}$ in Equation (5) and the edge computation time $T_{n}^{M}$ in Equation (6), with both depending on the task division $\alpha_{n}$. $T_{n}^{L}$ increases with $\alpha_{n}$, while $T_{n}^{M}$ is inversely proportional to $\alpha_{n}$. We can say that at some point, they will have the same value, and the processing time of task *n* reaches the minimum. Then, we can calculate the value of $\alpha_{n}$ by solving the following equation:

(8) $$\frac{\alpha_{n}c_{n}}{f_{n}^{L}}=\left(1-\alpha_{n}\right)(\frac{s_{n}}{{\hat{r}}_{n}}+\frac{c_{n}}{f_{n}^{M}}).$$

After some mathematical calculations, we find the optimal value of task division $\alpha_{n}$ as follows:

(9) $$\alpha_{n}^{*}=\frac{f_{n}^{L}\left(s_{n}f_{n}^{M}+c_{n}{\hat{r}}_{n}\right)}{c_{n}{\hat{r}}_{n}f_{n}^{M}+c_{n}{\hat{r}}_{n}f_{n}^{L}+s_{n}f_{n}^{L}f_{n}^{M}}.$$

As can be seen in Equation (9), the task division $\alpha_{n}^{*}$ depends on the local and edge computational capabilities ($f_{n}^{L}$ and $f_{n}^{M}$, respectively), the size of the data $s_{n}$, and the number of cycles $c_{n}$ required to compute the task.

Then, the non-convex optimization problem in Equation (7a) can be rewritten as follows:

(10a) $$\begin{array}{cc} \min_{b_{n},f_{n}^{M}} & \max_{n}\frac{\alpha_{n}^{*}c_{n}}{f_{n}^{L}}, \end{array}$$

(10b) $$\begin{array}{cc} C_{1}: & \sum_{n=1}^{N}f_{n}^{M}\leq f^{max},f_{n}^{M}\geq 0, \end{array}$$

(10c) $$\begin{array}{cc} C_{2}: & \sum_{n=1}^{N}b_{n}\leq 1,b_{n}\geq 0. \end{array}$$

The objective in Equation (10a) is a nonlinear function of both $b_{n}$ and $f_{n}^{M}$ and is subject to the sum constraints in Equations (10b) and (10c). $b_{n}B$ corresponds to the number of subcarriers allocated to node *n* in the OFDMA-based network. In [14], the authors studied a binary offloading problem by considering adaptive bandwidth allocation and MEC resource allocation. The bandwidth allocation was based on the (integer) number of subcarriers, and the MEC CPU resources were real numbers. Their problem was a mixed-integer nonlinear program problem which was NP-hard. Similarly, the problem in Equation (10a) is also NP-hard.

### 4.2. Optimal Resource Allocation

Due to the complexity of the joint optimization of wireless resource allocation and MEC resource allocation in Equation (10a), we propose solving it iteratively with an effective algorithm called Partial Offloading with Relay and Adaptive Bandwidth allocation (PORAB). Instead of alternate optimization, relay selection and the parameter $\alpha_{n}^{*}$ are determined in advance. Then, PORAB optimally allocates the bandwidth for transmission and computation resources for processing each task at the MEC server.

Basically, PORAB is an evolutionary algorithm [30]. At each generation, the population is generated based on the characteristics of the previous generation. Then, the unfit candidates are removed, and the fittest candidates are selected according to their characteristics. New candidates are generated in the next generation to keep the size of the population constant. This process repeats from one generation to the next generation until we obtain the desired solution. PORAB optimizes the problem by maintaining the numerical characteristics of each candidate in the population. In this way, the actual population does not need to be maintained from one generation to the next.

As shown in Algorithm 1, we first initialize all the parameters of our optimization problem. Uniform random distribution is used to initialize the population of the first generation with random candidates (line 3). During each iteration, the algorithm computes the maximum in Equation (10a) as the fitness value of each candidate ω∈Ω (line 5). The best fitness value is recorded in $\delta_{\omega ,t}^{*}$ (line 6). If the difference between the optimal fitness values of the current and previous iterations is less than a parameter (i.e., $|\delta_{\omega ,t}^{*}-\delta_{\omega ,t-1}^{*}|\leq \epsilon$), then it is said that the algorithm has converged, and no further computation is required. Otherwise, the $s=\left|\Omega \right|S_{p}$ best fit solutions, where $S_{p}\in \left(0,1\right)$ is a selection probability, are selected as $\bar{\Omega }$ (line 10).

For each dimension $\omega_{n}$, the mean ($\mu_{n}$) and standard deviation ($\sigma_{n}$) are computed over $\bar{\Omega }$, and they form the vectors μ and σ, respectively (lines 11–12). Next, new candidates are generated while a premature convergence is avoided (lines 13–20). Using the mean and standard deviation of each dimension v∈ω, a window $\left(v^{low},v^{high}\right)$ is calculated, where $v^{low}$ and $v^{high}$ represent the lower and upper bounds of dimension *v*, respectively. If its window size $v^{high}-v^{low}$ is too small, being less than the predefined value $\gamma_{v}$, then its window is reset to $\left(\omega_{n}^{l},\omega_{n}^{u}\right)$. This process is repeated for all dimensions of ω. Then, with the new windows, a new population is generated for the next generation using uniform random distribution.

In each iteration of Algorithm 1, the computation mainly takes place in three places. First, there is the computation of the fitness value $\delta_{\omega }$ for each sample ω∈Ω (line 5), which is O(N·|Ω|). Next, the best *s* solutions required to sort the population are selected, and the complexity is O(|Ω|log(|Ω|)) (line 10). Then, the time for updating the population is O(N·|Ω|). Assume that the maximal number of iterations is *L*. Accordingly, the overall computation cost is O(L·|Ω|·max{N,|Ω|}).

**Algorithm 1:** Partial Offloading with Relay and Adaptive Bandwidth Allocation (PORAB)

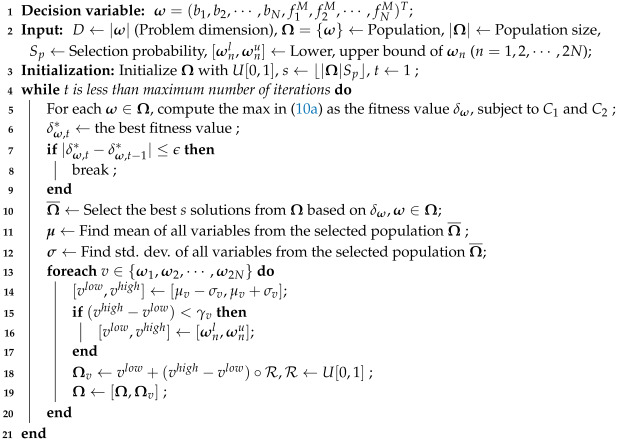



**Figure 3:**
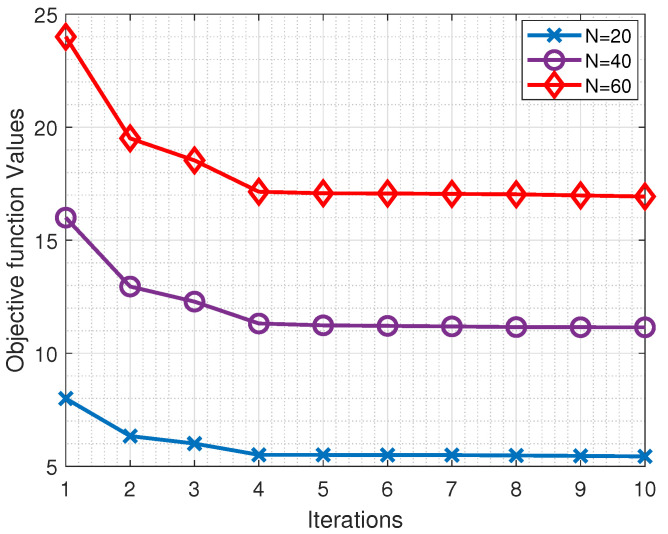
Algorithm convergence graph.

The convergence graph of the proposed algorithm is shown in Figure 3. The results show how the algorithm converged with iterations under different numbers of nodes *N*, where N∈{20,40,60}. Here, we assumed a block fading channel was between the nodes and AP, which means that the transmission rate was constant for the entire period *T*. We observed that as the number of iterations increased, the algorithm gradually converged to an optimal solution. The converged result of the algorithm increased with the number of nodes *N* because more nodes led to more computational requirements at the MEC server.

## 5. Simulation Evaluation

In this section, the performance of the proposed method is evaluated via simulation in the MATLAB environment.

### 5.1. Simulation Setting

The simulations were performed by using the parameters shown in Table 1. We assumed that *N* IoT nodes were randomly distributed in the coverage of an AP, and the channel between a node and the AP was constant within the transmission time.

The proposed PORAB method has both relay selection and adaptive bandwidth allocation functions. It was compared with other methods, namely Partial Offloading with neither function (POBase) [27], Partial Offloading with only Relay selection (POR), and Partial Offloading with only Adaptive Bandwidth allocation (POAB). Here, both POR and POAR implemented one of the functions of the proposed method. In this way, the comparison between PORAB and POR or POAR provided an ablation study.

In the evaluation, we used the transmission rate and task computation time as the evaluation metrics. The latter was the maximal execution time of all tasks, each of which was the maximum of local processing time $T_{n}^{L}$ and the MEC offloading time $T_{n}^{M}$ (including both transmission delay and the processing time at the MEC). Because each node had only one task, the number of nodes equaled the number of tasks.

### 5.2. Simulation Results

**Figure 4:**
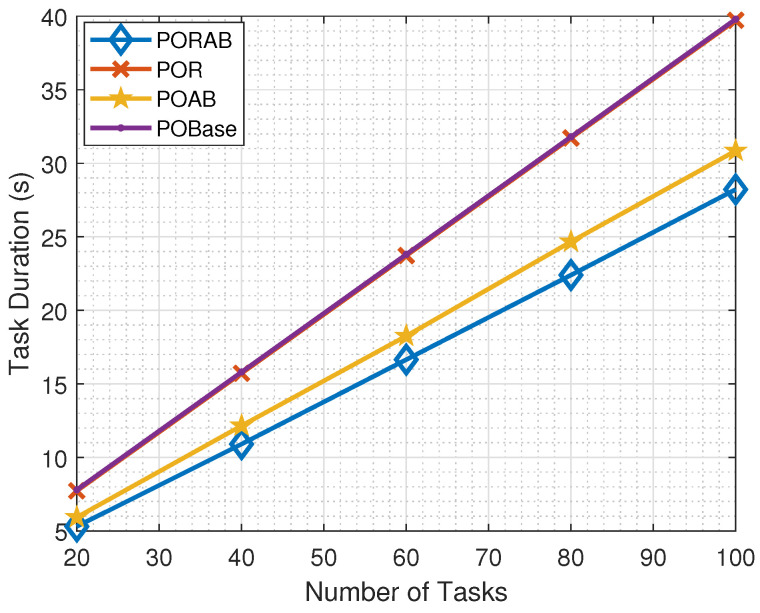
Variation of task computation time with the number of tasks.

Figure 4 shows how the task computation times in different methods changed with the number of tasks in the network. Generally, in all methods, the task computation time increased with the number of tasks. This was because the computation resources at the MEC server were fixed, and each task would have fewer resources when there were more tasks, which increased the edge computation time.

When each task was allocated equal bandwidth, using a relay did not help much, and the task computation time was almost the same for POBase and POR. When adaptive bandwidth was used, both PORAB and POAB achieved better performance than POBase and POR. In addition, the effect of the relay became obvious. PORAB outperformed POAB, and the improvement in task execution time increased with the number of tasks.

**Figure 5:**
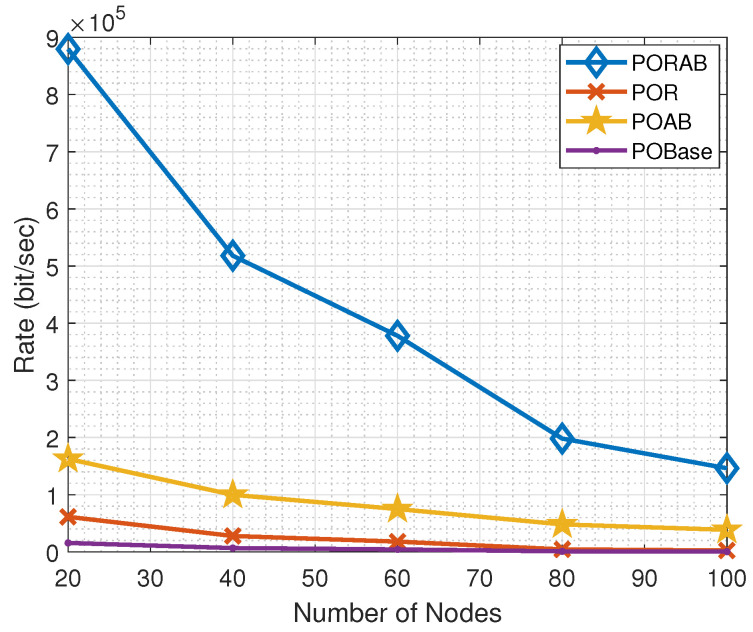
Variation of transmission rate with the number of nodes.

In our model, we optimally allocated the bandwidth, and we can see in Figure 5 that the average data transmission rate improved. For a small number of nodes, the data transmission rate was high. When the number of nodes increased, the data transmission rate decreased, but our proposed PORAB algorithm outperformed POAB.

**Figure 6:**
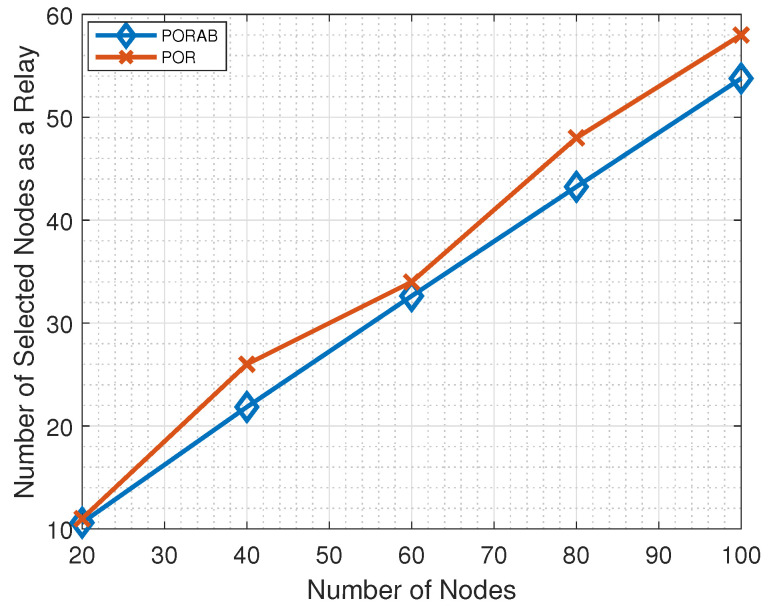
Variation of the number of selected relays with the number of nodes.

Figure 6 shows the number of nodes selected for the relays. Generally, the number of relays increased with the number of nodes. Using adaptive bandwidth, the number of relays can be reduced in PORAB, which helps to reduce the burden of the nodes.

Table 2 shows how the number of selected relays varied with the number of nodes. When N=20, the number of nodes selected as relays was 11 in POR but reduced to 10 in PORAB. Similarly, when N=40, the number of nodes selected as relays was 26 and 22 in POR and PORAB, respectively. We can see from Table 2 that the number of relays increased with the number of nodes.

**Figure 7:**
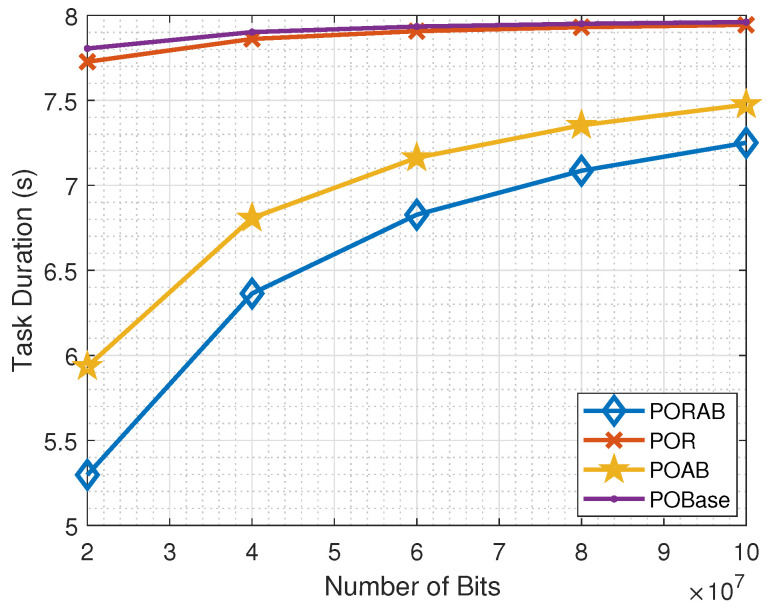
Variation of task computation time with the volume of tasks in terms of bits.

Figure 7 shows how the task computation time varied with the volume (size) of the tasks. Generally, the task computation time increased with the volume of the tasks in all methods. However, the proposed PORAB method achieved the best performance. When the volume of data was low, the required amount of computational cycles was small, and the task computation time was small. In the partial offloading, a part of the task was computed locally, and the remaining part was computed on the MEC server. The time required to compute a part of a task was less than that required to process the whole task on the MEC server. As a result, the proposed PORAB method minimized the delay and was expected to meet the requirements of fifth-generation wireless communication systems.

**Figure 8:**
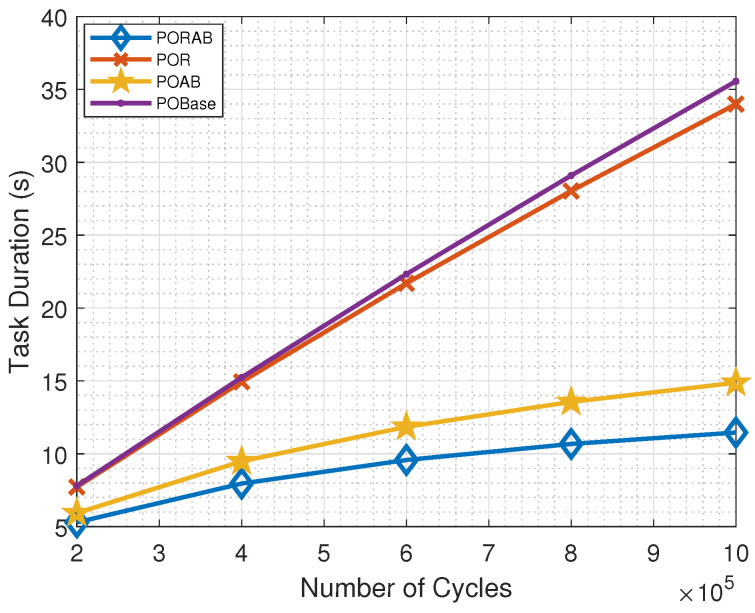
Variation of task computation time with the number of cycles required to process a task.

Figure 8 shows that the task computation time increased with the number of cycles required to process each task, but the trend was different. With adaptive bandwidth allocation, the transmission could be made more efficient, and more time could be used for processing at the MEC server. By using relays, PORAB further improved the performance compared with POAB.

The above results indicate that both relays and adaptive bandwidth allocation helped to reduce the task computation time. Adaptive bandwidth allocation fine-tuned the transmission rates and showed a very obvious effect, regardless of the use of relays. In comparison, the effect of the relay was larger when used together with adaptive bandwidth allocation.

## 6. Conclusions

In this paper, to solve the potential transmission delay caused by the low transmission rates of nodes far from the AP in multi-task partial offloading, we have suggested using relay selection and adaptive bandwidth allocation. We minimized the overall task computation time, jointly considering relay selection, bandwidth allocation for tasks, task division between nodes and the MEC server, and resource allocation for tasks at the MEC server. PORAB, as an evolutionary algorithm, was proposed to iteratively solve the problem. The simulation results show that the proposed method effectively reduced the task computation time compared with other benchmark methods. 

## Figures and Tables

**Table 1:** Main notations in the model and their default values [23,29].

Parameters	Descriptions	Values
*N*	Number of nodes	100
*B*	Overall bandwidth	2 MHz
$b_{n}$	Percentage of bandwidth for task *n*	By algorithm
$c_{n}$	Number of processing cycles of task *n*	[0.2,0.4,⋯,1] M cycles
$s_{n}$	Data size of task *n*	[20,40,⋯,100] M bits
$f_{n}^{L}$	Comp. resource of node *n* for task *n*	1 M cycle/sec
$f_{n}^{M}$	Comp. resource for task *n* at MEC server	By algorithm
$f^{max}$	Overall comp. resource at MEC server	25 M cycle/sec
$\alpha_{n}$	Percentage of task *n* for local processing	By algorithm
$P_{max}$	Maximum energy of a node	2 joules

**Table 2:** Variation of the number of selected relays with the number of nodes.

*N*	20	40	80	100
POR	11	26	48	58
PORAB	10	22	43	53

## Data Availability

Not applicable.
